# Developing the aquaticity level in healthy adolescents. A randomized control study

**DOI:** 10.3389/fspor.2024.1437338

**Published:** 2024-12-24

**Authors:** Danae Varveri, Christina Karatzaferi, Elizana Polatou, Giorgos K. Sakkas

**Affiliations:** Department of PE and Sports Science, School of Physical Education, Sport Science and Dietetics, University of Thessaly, Volos, Greece

**Keywords:** swimming ability, water safety, drowning [prevention and control], health, aquaphobia

## Abstract

Aquaticity is an important parameter of human aquatic performance and behavior and can be objectively assessed by the aquaticity assessment test. Low aquaticity score can unveil a person's high risk in the water while it could dictate the specific characteristics that need to be addressed or developed for improving water competence.

**Aim:**

The aim of the current study was to assess whether human aquaticity can be developed by systematic exercise and which type of training is more effective in improving aquaticity score.

**Methods:**

Twenty healthy untrained, high school students (8M/12F, 16.5 ± 0.7) participated in the study after obtaining parental consent. Participants were screened for their aquaticity level using the Aquaticity Assessment Test (AAT) and randomly divided into two groups: Group A (4M/6F, 16.3 ± 0.8) completed a classical swimming training program, while Group B (Aquaticity) (4M/6F, 16.8 ± 0.5) completed the aquaticity intervention program. Both interventions lasted for two months (3 workouts per week, lasting 60 min per session) while participants assessed before and after the training period using the same testing protocol and evaluators.

**Results:**

Aquaticity score was improved after training by 13% (13.23 ± 6.88%) for Group A (Swimming training) and 26% (−26.6 ± 10.40%) for Group B (Aquaticity training) (*p* = 0.004). In Group A (swimming), 7 out of 10 tasks were improved significantly compared the pre-values (*p* < 0.05) while in Group B (aquaticity) 10 out of 10 shown significant improvements compared to pre-training values. Interestingly, the magnitude of change between the two groups was statistically significant in 5 out of 10 tasks (tasks 2, 3, 7, 9, 10) implying a higher magnitude of improvements in the aquaticity intervention group compare to swimming group.

**Discussion:**

Aquaticity can be developed and improved when a specific training program applied. Essential to water competence aquaticity skills can be advanced using simple aquaticity training games that can improve water confidence and reduce drowning related accidents.

## Introduction

Aquaticity is an important parameter of human performance and behavior. “Aquaticity is the capacity of a terrestrial mammalian organism to function and habitualise in the aquatic environment (1). The level of aquaticity depends on mental and physical characteristics and can be improved by frequent exposure to the water element especially when full body immersion training is taking place (2–4). Τhe way that humans interact with water varies and depends on prior experience, familization with the water element, prior trauma (physical or mental) as well as the current state of mental health, leading either to water confidence or to water-phobia and/or related phobias (5, 6). There are three types of aquaticity. “Physical aquaticity” where is the ability of a person to interact with the water element with no support from any technical equipment while “technical aquaticity” is the ability of a person to use various tools and equipment in water. “Interactive aquaticity” is when a person uses rafts, boats and other carriers in or under the water (1). Aquaticity offers the characteristics that humans need in order to effectively adopt and function within the water environment. Effective function within the aquatic environment is also translated into “safety” especially in young children and toddlers. Despite the numerous precautions and safeguards put in place, drowning remains a leading cause of unintentional injury-related preventable death among children, particularly toddlers and young children. Children aged 1–4 years old are at heightened risk for injury due to their physical capabilities outpacing their cognitive understanding of potential dangers (7).

Our group has developed and validated an aquaticity assessment test (AAT) for the evaluation of human's potentials in the water (8). The AAT can be used as a water competence assessment tool, which screens motor and cognitive-perceptual skills in the water (e.g., working memory, eye—hand coordination underwater, shape and color detection without the use of swimming goggles). At the same time, the AAT can be used as a talent identification test when an individual achieves a high score. Anecdotal reports from coaches support the notion that classical swimming training can improve aquaticity level however there are no scientific evidence to support such statements. The only study that has examined the efficacy of a specialized school-based aquatic intervention in improving aquaticity in healthy untrained children (8–9 years old) comes from our group where it has been shown that even nine training sessions (9 weeks, 1 day week) incorporated into the physical education course of the school curriculum, improved the total score of the aquaticity testing protocol by 48% (9). It seems that aquaticity can be trained and improved when specific training routines applied; however, it is not known whether this is also achievable with classical swimming training and to what extent. Therefore, the aim of the current study was to assess whether human aquaticity can be developed by systematic exercise and which type of training is more effective in improving aquaticity score. The objectives of the study are to compare classical swimming training focusing in performance to an aquaticity-specific training program focusing in aquatic competency. We hypothesized, that any interaction with the aquatic environment will improve overall aquaticity, however, specific training aiming to improve the capacity to function and habitualise in the aquatic environment will be more effective.

## Materials and methods

### Participants

Twenty high school students (8M/12F, 16.5 ± 0.7 years) were screened for the current study. Medical clearance was obtained for all participants including full cardiac assessment. Inclusion criteria were: parental consent, attending high school classes, ability to swim or float for 25 meters using any style and the medical clearance. Exclusion criteria were currently participating in swimming clubs attending swimming training classes, aquaphobia, inability to float or infection and musculoskeletal injuries interfering with the swimming activities and also vision or ear problems. Participants were screened for their aquaticity level using the Aquaticity Assessment Test (AAT) as described previously (8) and randomly divided into two groups matching carefully age, gender and aquaticity score. Group A was the “Swimming” intervention group while Group B was the “Aquaticity” intervention group.

Study approval was obtained from the University of Thessaly Research Ethics Committee (GR518/29/03/2012). Information letters and consent forms requesting parental permission were sent to potential participants’ homes while those who returned signed were screened for the eligibility criteria.

### Study design

A two months intervention using either classical swimming or aquaticity training included in the current study design. Group A (Classical swimming group) was consisted of 10 subjects (4M/6F, 16.3 ± 0.8) and participated in a classical swimming training program lasting for 60 min per session, 3 workouts per week for two months (please refer to Tables 1–3 for details regarding the components of the programs). Group B (Aquaticity group) consisted of 10 subjects (4M/6F, 16.8 ± 0.5) and participated in an aquaticity intervention program lasting for 60 min per session, 3 workouts per week for two months (please refer to Tables 1–3 for details regarding the components of the programs). Participants were re-assessed after the 2 months training period using the same set up and evaluators. All training sessions were supervised by the same qualified swimming instructor taking place on separate days and times for each group. Adherence to the training program was 98% and 97% for Aquaticity and classical swimming groups respectively.

**Table 1:** Intervention programs -workout number: 1st, 4th, 7th, 10th, 13th, 16th, 19th, 22nd.

	Intervention programs	
	Macro cycle 1, week 1–8	Pool length: 25 m—pool depth: 1.60–2.85m Water temperature: 27C
Workout number: 1st, 4th, 7th, 10th, 13th, 16th, 19th, 22nd	Session duration: 60 min	Program structure: warm up-main set-secondary set-cool down
	Aquaticity	Classical swimming
Training zone: aerobic	Length of session 1,600 m Effort up to 70% Heart rate 120–175 bpm, Perceived exertion: comfortable & somewhat uncomfortable	Length of session 2,500 m Effort up to 70% Heart rate 120–160 bpm Perceived exertion: comfortable & somewhat uncomfortable
Training aims	Physical efficiency and technique development: 1. Swimming on surface (partial immersion). different swimming styles (FS, BK, B S, BF, sculling, lifeguard swim, dolphin kick, egg-beater kick, balance and streamline position in each side, prone and supine body position. 2. Underwater training stimulus (total immersion): relaxation techniques as a preparation for breath holding, how to low heart beats with efficient breathing patterns: and relax body tension: short static apnea: u/w buoyancy control: dynamic apnea nofins (underwater swimming-pull down breaststroke), hydrodynamic position and gliding- underwater push-off from the wall.	Aerobic development, stroke technique enhancement-drill progressions for all four competitive strokes, develop core elements of body strength and control, enchase the feel of the water, kick and pull training. Main set -Objectives: volume- speed combination & work on technique (i) improving aerobic capacity, aerobic muscular endurance and (ii) improving anaerobic muscular endurance, improve fitness and efficiency on taking fewer strokes per length of the pool, turns, stroke & kicking technique.
Drills: main & secondary set	Warm up: continuous swim 400 m (200 m FS- 200 m any style). Main set: 4 × 50 m focus on kicking [50 m side swim sessors kick, 50 m lifeguard side swim (back hand straight up), 50 m butterfly kicks on side (left & tight), 50 m egg-beater kick] 4 × 50 m stroke technique (50 m FS- BS-BrS- one arm BF) rest: 30 s 4 × 50 m on supine body position [50 m breaststroke kick, 50 m supine position, sculling with the head/scaling with the legs: 50 m backstroke kick: 50 m egg-beater kick] 4 × 50 m swimming (FS, BS BrS, BF) rest 30 s 16 × 25 m dynamic apnea progressions [4 × max gliding distance after push — off from the wall: 4 × 12.5 m Dyn no-fins swimming-pull down breaststroke, 4 × 12.5 m Dyn only breaststroke kick, 4 × 12.5 m Dyn only arm stroke] rest: 12.5 m swim down the rest distance to the wall + 1 min rest. 1. Relaxation & breathing techniques a. floating positions b. diaphragmatic breathing b. Hanging static apnea technique 2. Static apnea 6 sets [3sets on prone floating position, 3 sets hanging from the wall -at max depth of the pool] rest 2 min Cool down: 100 m any stroke	Warm up: 600 m low intensity swim 200 m FS (rest 15sec) 200 m IM kick development, 200 m personal stroke. Main set: 4 × 200 m FS on 3.00 min, with bilateral breathing (the 2nd and the 4th breathing every three, five, seven). Pyramid 25 m, 50 m, 75 m, 100 m, 75 m, 50 m, 25 m (25 m BF, 50 m BK, 75 m BS, 100 m IM). 8 × 50 m on a 1.05 min send-off, personal stroke Secondary set: work on turns for all strokes Objectives: develop technique points (approach towards the wall, fast turn or rotation if it is two handed touch, dynamic push off and exploding through the knees and hips to maintain streamline position from the arms through to the pointed toes, hydrodynamic head position, fast & vigorous dolphin kicks ensuring maintenance underwater streamline position, practice turn both ways L&R). Cool down: 5 min. 200 m Swim- down. any stroke.

**Table 2:** Intervention programs -workout number 2nd, 5th, 8th, 11th, 14th, 17th, 20th, 23nd.

	Aquaticity	Classical swimming
Workout number: 2nd, 5th, 8th, 11th, 14th, 17th, 20th, 23rd	Session duration: 60 min	Program structure: warm up-main set-secondary set-cool down
Training zone: aerobic anaerobic lactic	Length of session 1,500 m Effort up to 80% Heart rate 120- 175 bpm Perceived exertion: comfortable and uncomfortable	Length of session 2,000 m Effort up to 100%/Heart rate 120–190 bpm Perceived exertion: fast but comfortable (short sprints)
Training aims	1. Development of strength and endurance in the water: Develop elements of various movement pattern, for body strength and control (swimming, sculling, scooping, kicking, egg-beater, u/w swimming & buoyancy) 2. Physical efficiency in the water 3. Orientation and habituation: zic- zac swim, stop and go, swim-dive-resurface- swim, roll swim front and back 4. Floating training: stationary (back layout with or without scull, side layout, tub and tuck, rolls, oyster, water wheel, logroll)	Resistance training, develop core body strength and stability, kick and pull training to increase stroking power and short sprints. Objectives: (i) Build resistance kick speed kick with fins technique -based pulling land resistance pull training. (ii) Develop pure speed (iii) technique: work on starts
Drills: main & secondary set	Warm up: 400 m continuous swim 200 m FS (rest 15 sec), 200 m kicking with no kickboard- any kick style (rest 15”) Main set: 15 × 30 sec/rest 15 sec. (l) Stationary Treading Water: egg- beater/BrS kick. (2) Stationary Sculling: Flat sculling position to butterfly-turn and streamline position (L&R)/Flat Sculling position to backstroke-turn and streamlining on the back. (3) Front and back somersault with a tight tuck (4) Reverse body position: vertical sculls. (5) Vertical push offs- streamline Jumps + turns (FS/BK). (6) Water wheel. Distance swimming different movement pattern: 2 × 50 m/rest 15 sec/egg- beater distance swim: prone and supine position (double arm sweep in, sweep out)/BS kick distance swim: prone and supine position/Head first horizontal scull on back Sculling with legs leading/Short swim distance- both high velocity + changing directions (L&R/F&B)—intense bursts of activity, lasting less than 15 s-followed by recovery (lower-intensity swim) (head-high FS)/Scooping-A clean catch and ability of hold the water, helps to maximize stroke efficiency. (Propeller- face remains on surface) supine position Buddy-Up Partner Exercises: Entry-Approach the partner at 25 m distance point-Tow back/Giant Stride entry—approaching the partner performing head high breaststroke—Towing (Kickback, Inverted Breaststroke Kick with two Arms)/Grab start entry—approach FS no breath — towing back eggbeater kick/Entry- duck dive — approach the partner underwater- tow back side stroke Objectives: Teamwork/Cognitive function: concentration, adaptability, problem solving in the water. Developing psychological and emotional conditioning in the water, emotional stability, concentration, confidence, developing water awareness and determination—will of power under difficult conditions. Drills: l.5 min, Relay 2 teams- 25 m—swimming with eyes closed- requires a pair of goggles that have been blacked out, slow swimming any stroke 2. Relay 2 teams- 25 m—swimming with a cotton bath rope—one per team (take it off—give to teammate and wears it on- all process in the water) 3. Relay 2 teams- 25 m swim with fins by holding a kettlebells (l kg) Cool down: 250 m swim down any stroke	Warm up: low intensity swim 200 m FS (rest 15 sec), 200 m IM kick development -count the number of kicks done every 50 m (re 15 sec), 200 m personal stroke- negative split. Main set—Objectives: (i) Build resistance kick/’speed kick with fins technique -based pulling land resistance pull training. (ii) Develop pure speed a. 4 × 100 m FS swimming with paddles on a 1.10 min send off. b. 8 × 50 m main stroke kick with fins, negative split. c. 16 × 25 m maximum speed (l FS I personal stroke) rest 1.30 Work on starts for all strokes Objectives: development of grab starts and backstroke start Cool down: 5 min, 200 m Swim- down

**Table 3:** Intervention programs -workout number 3rd, 6th, 9th, 12th, 16th, 19th,21st, 24nd.

	Aquaticity	Classical swimming
Workout number: 3rd, 6th, 9th, 12th, 16th, 19th, 21st, 24nd	Session duration: 60 min	Program structure: warm up-main set-secondary set-cool down
Training Zone: Anaerobic endurance	Length of session 1,600 m Effort up to 90% Heart rate 120–200 bpm/Perceived exertion: uncomfortable	Length of session 2,200 m Effort up to 95%/Heart rate 120-190 bpm Perceived exertion: hard work
Training aims	a. Physical efficiency in the water b. Underwater intensive training: breath hold ability, underwater buoyancy, Dyn with fins c. Cognitive function: adaptability -mind tasks- team work	Speed training-working on the ratio, stroke efficiency, hydrodynamic body position, develop the feel of the water in sprints, momentum, gliding, body roll
Drills: main & secondary set	Objectives: Combination surface and underwater swimming (with fins)- Hypercapnic training (increased levels of C02) -hydrodynamic position (head & body)- surface breathing preparation — recovery breathing- conscious control of the mammalian diving reflex Drills: 4 × 50 m [25 m FS with fins/25 m u/w FS kick with fins] rest I min 4 × 50 m [25 m on surface BF with fins on apnea 125 m u/w BF kick with fins] rest 1 min 4 × 25 m Static Apnea on the wall and directly swim u/w FS kick with fins, rest 1 min (whistle sign) 4 × 25 m u/w BF kick with fins and directly Static Apnea (or count to 10) rest 1 min 8 × 25 m u/w sprint any stroke (with or without fins) rest 1.5 min. Objectives: Dive technique, Apnea ability, underwater buoyancy control, Cognitive function—develop awareness being underwater, underwater vision training, mind challenges—develop brain activity Drills: no swimming goggles, no fins -Underwater team Game 2 teams Equipment: 2 Underwater Writing Dive Slates (1m × 1 m) + pencils, 40 glass marbles, 2 padlocks and multiple keys [one lock find the right key], 7 waterproof plastic cards [picture I fruit + I number], stopwatch • Wins: The team that completes all 4 tasks [best time and less mistakes] DESCRIPTION: 1. “Draw & Write—underwater”, one dive slate per team is immersed in the max depth of the pool (from—2 m up tp-2.80 m depth), all players have to draw a triangle & a circle inside and under the draw have to writ: the word “team” Score: 5 points when all draws & names are clear [Drawn mindlessly each doodle & careless handwriting each scribble. 2. “Find the right key! one lock multiple keys”. At the deep side of the pool (−2.00 m) approx. 1 m shallower depth than task l. one padlock for each team and multiple keys are immersed. Each participant dives, chooses one key and tries to unlock the padlock. When that is achieved the team continuous to the next challenge. Score: time-oriented score 3. “Match the cards” (depth — 2.00 m). One card is placed out of the water (showing one fruit and one number), this is the same card for both teams. Participants have to dive and pick from 10 immersed cards the one that shows the same picture. Score: time-oriented score 4. “Yellow glass marbles”, (approx. in −150 m depth), 40 colored glass marbles are immersed in the bottom. Every participant has to find one yellow marble and place it at the deck. Score: 5 if all team members find the yellow marble, -l point for every missing marble and I extra point team player, when the task is achieved on the first try {Cool down: 100 m any stroke}	Warm up: low intensity swim 200 m FS (rest 15 sec), 4 × 100 m progression drills in all four strokes (rest 15 sec). Objectives: teaching swimmer the use Of one's entire body when swimming at speed- “how to hit your top speed”, develop muscle strength, fast shape ring effect rapid improvement a. 2 × 200 m b. 4 × 100 m c. 8 × 50 m FS, max speed (rest 2 min) 1. Relays 4 × 50 m (free & medley) 2. work on finishes for all strokes and relay takeovers Objectives: teamwork, enjoyment, development of finishes Cool down: 5 min. 200 m Swim- down

### Aquaticity assessment test—AAT

The AAT used to score the aquaticity level of the participants before and after the intervention. The test is composed of 10 tasks required to be completed by the participants during the assessment (8). Briefly, the tasks assessed the following parameters: (1) Surface stationary floating and balance control, (2) Breathing control, (3) Underwater hydrodynamic position and gliding, (4) Surface freestyle swimming technique, (5) Physical adequacy (5 min continuous swimming, any style), (6) Treading water- egg beater kick, (7) Underwater vision and cognitive-perceptual skills, (8) Underwater sound detection, (9) Underwater breath hold swimming (dynamic apnea) and (10) Expiratory—breath out diving. Each task was scored from 0 (fail) to 5 (excellent). For each task, participants could achieve a score from 0 to 5 depending on the level of adequacy they demonstrated. Examiners assigned points (in 0.5 step increments) based on the fidelity of performance to the given instructions, repetitions achieved, duration of sustained performance, and other criteria. Participants who achieved a score of 4.5 could try to achieve an excellent score (5 points) by completing a variation of the task with advanced complexity after a one-minute break. The highest overall score that could be achieved by a single subject is 50 points. The AAT identifies participants with high (≥43.3), medium (from 23.8 to 43.2) and low (≤23.7) aquaticity level and therefore low physical adequacy in the water and increased risk for a potential drowning event.

To give a better insight into the purpose of the study parameters were also analyzed based on (1) Breathing Control (Basic vs. Advanced) and (2) Aquatic Skills (Motor vs. Cognitive-Perceptual). This further analysis helped to better understand which tasks of the testing protocol, developed more by the swimming training and which from aquaticity training.

### Specifically

(1) Breathing Control
  (a) Basic Breathing Control refers to tasks in which foundational breathing techniques need to be performed. These involve either partial immersion (face submersion) or total body immersion up to −1 m depth during exhalation (tasks 2–5, 8).
  (b) Advanced Breathing Control refers to tasks in which breath-holding is voluntarily performed along with underwater buoyancy control while coping with cognitive-perceptual challenges at depths from −1.5 m to −2.8 m (tasks 7, 9, 10).
(2) Aquatic Skills
  (a) Aquatic Motor skills refers to tasks in which stationary and propulsion skills are performed (tasks 1, 3–6).
  (b) Aquatic Cognitive-Perceptual Skills refers to tasks in which an individual's ability to perform multiple mental processes underwater is assessed. This includes working memory, eye-hand coordination, shape and color recognition, sound detection underwater (tasks 8–10).

Categorization of the ten tasks is given in Table 4.

**Table 4:** Categorization of tasks based on breathing control or aquatic skills.

	Aquatic breathing control		Aquatic skill types	
	Basic	Advanced	Motor skills	Cognitive-perceptual skills
Category of Task	2	7	1	7
	3	9	3	8
	4	10	4	9
	5		5	
	**8**		**6**	

Task description: (1) Surface stationary floating and balance control, (2) Breathing control, (3) Underwater hydrodynamic position and gliding, 4) Surface freestyle swimming technique, (5) Physical adequacy (5 min continuous swimming, any style), (6) Treading water- egg beater kick (7) Underwater vision and cognitive-perceptual skills, (8) Underwater hearing, (9) Underwater breath hold swimming (dynamic apnea) and (10) Expiratory—breath out diving.

Bold indicates statistical significant values.

### Facilities

Both intervention programs were held in a private's school swimming pool center, in Athens, Greece. The data analysis carried out at the Lifestyle Medicine Laboratory at the School of Physical Education and Sport Science, at the University of Thessaly, Greece.

### Intervention

Training details are summarized in Tables 1–3.

#### Group A—classical swimming group

The participants in Group A followed a classical swimming training intervention using modern training methods that are based on both hydrodynamics and biomechanics (10). Classical swimming is a technique-driven sport, and each session prepares participants for the best swimming performance—speed, endurance, and technique perfection—in the four competitive swimming strokes: freestyle, backstroke, breaststroke, and butterfly. The training goal of the classical swimming program (as with any swimming training program at this level) was to develop aerobic and anaerobic capacity, enabling participants to eventually cover racing distances at maximum speed. In addition, the training program was also aimed at developing technical elements such as floating ability, hydrodynamic body position, bilateral breathing pattern and coordinated body movements, effective kicking action, and quality stroke mechanics, which will allow for more efficient propulsion and performance. Therefore, the drill progressions started with the basics and linked to full movements through repeated instructions and visual feedback. Since the goal of a competitive swimmer is to cover a given distance in the minimum amount of time, we also worked on starts, turns, and finishes. The percentage of varying training intensities when averaged out is: aerobic training 60%, aerobic/anaerobic 20%, in water strength training 15% and anaerobic lactic 5%. Each training session was composed by warm up, main sets and cool down. The total swimming distance covered in each session was 2,000–2,500 m depending on the training objectives (Tables 1–3).

#### Group B—aquaticity group

The participants in Group B followed an Aquaticity Development Program (ADP). The ADP is free from competitive strategies and uses task-based exercises to develop participants’ perception and adaptability in the aquatic environment by integrating cognitive-perceptual functions with physical and visual-spatial training. We applied training stimuli both on the surface, with the body partially immersed, and underwater, during total immersion. We used the interaction of motor and cognitive-perceptual training as a strategy to enhance participants’ physical efficiency in the water, strength, and technique, as well as breath-holding ability and sensory adaptability. The aquaticity intervention included training drills for:

1. Physical efficiency and adequacy

Aquaticity intervention used five training methods: aerobic, anaerobic, resistance, hypoxic and hypercapnic (10) and particularly applied various stimuli including:

(a) Continuous swimming on the surface (aerobic ability) similarly to the swimming group.
(b) Strength and endurance with water exercises (pulling, sculling, scooping, kicking, treading water, underwater sprints with fins, buddy exercises) (11).

2. Technique—Aquatic Motor Skills
  (a) Propulsion skills: In aquaticity program, different kinds of propulsion skills (strokes, kicks, sculling, and eggbeater) are taught and practiced, extending the participants’ movement patterns in the water and enhancing their effective propulsion techniques (11, 12). Sculling skills were particularly practiced so that participants could learn to ‘feel’ the mass of the water. The feel of the water refers to the participant's intuitive ability to effectively handle the water during stationary or propulsive movements. The sense of touch in every stroke and kick, along with the sensation of moving pressure, leads to advanced stroke technique and greater expertise (10). In challenging exercises, propulsion skills were combined with abilities such as breath-holding, towing, lifting submerged objects, swimming with clothes, and other tasks.
  (b) Stationary skills—floating/buoyancy control/orientation: These skills are fundamental for water competence and safety. The aquaticity program developed these skills in different water layers (shallow, middle, deep) and by interchanging directions (forward-back, left-right) in combination with multiple movement patterns (swimming, floating, diving) (8) and through different body positions (prone, side, back, vertical, reverse vertical). In the most challenging exercises, stationary skills were implemented by combining breath-holding with mental-based challenges (e.g., visual detection—diving to resurface a specific object from a variety of similar submerged objects or diving to write your name on an underwater slate).
(3) Breath hold and diving ability:

This type of training is based on hypoxic and hypercapnic stimuli during both static and dynamic apnea (9). In hypoxic training participants developed tolerance in low levels of O_2_ and during hypercapnic training participants developed tolerance in high levels of CO_2_. None of the participants were forced to hold their breath more than they felt comfortable.

Relaxation techniques are essential elements for breath-holding ability and breath control. Relaxation ability in the water can be developed by training breathing patterns, floating skills, and buoyancy control. The first step included learning conscious breathing. The participants developed proper breathing preparation before every breath-hold session (static or dynamic apnea). The second step involved learning diaphragmatic breathing. This deep breathing skill is characterized by the expansion of the abdomen rather than the chest when inhaling. The participants became familiar with it and practiced patterns of ‘deep inhale-hold-slowly exhale’ to get accustomed to apnea techniques. The third step was to develop various free diving techniques, including the duck dive, the sense of being vertical, bottom turn and swimming with the least water resistance possible. Free diving is a multi-skilled parameter in the aquaticity program. The practice of apnea and free diving skills, aimed to promote, the participant's water awareness and the habituation in the underwater environment. Breath-holding interacts with various other skills, such as sensory adaptability, ear equalization, orientation, underwater propulsion, underwater buoyancy, mental clarity, and emotional stability. In the aquaticity program diving skills aimed to develop: 1. underwater motor skills and physical stamina, 2. cognitive-perceptual function underwater, 3. psychological conditioning (e.g., ability to cope, problem solving, emotional stability underwater), 4. soft skills (e.g., team work, willingness to learn new skills, confidence).

(4) Cognition and Perception underwater

Aquaticity training aims to develop cognitive-perceptual skills and activate sensory functions underwater, while also increasing enjoyment and water safety. Following training practices have been applied to foster senses adaptability in the participants such as underwater vision tasks and sound detection challenges. Specifically, various creative challenges without goggles aimed to develop underwater visual skills, such as the ability to detect and estimate the shape, size, color, and distance of submerged objects, as well as eye-hand coordination skills (13). Underwater hearing stimuli were also applied in the aquaticity program, aiming to develop the participants’ sound localization. During the ADP, participants practiced underwater games, either individually or in small teams. This type of training aimed to develop physical endurance, flexible and strategic thinking, confidence, determination, problem-solving skills, emotional stability, and overall adaptability in the aquatic setting (11).

Each training session was composed by warm up, main set, secondary set and cool down. The total swimming distance covered in each session was approximately 1,600 m depending on the training objectives (Tables 1–3).

### Statistical analysis

Two data analysis were conducted, each addressing one of the purposes of the study. The first data analysis aimed at comparing Aquaticity test score between Swimming Group A and Aquaticity Group B at baseline and after the intervention period (Pre and Post). Additionally, the tasks of the testing protocol (AAT) were categorized into Basic Breathing Control (Tasks: 2–5, 8) Vs Advanced Breathing Control (Tasks: 7, 9, 10) and into Aquatic Motor Skills (Tasks: 1, 3–6) vs. Aquatic Cognitive Skills (Tasks: 7, 8, 10). The scores on these tasks were averaged in order to produce an overall score for each participant for each Category of task. Due to the size of data, no complex statistical design was permitted, therefore, 2 variables per time were compared. For comparing the differences between groups an independent *t*-test was used while differences within groups assessing pre and post values were assessed using a paired *t*-test. Second data analysis was conducted within groups and between the ten tasks performed from the two groups pre and post intervention using a paired *t*-test while Delta changes were compared using an independent *t*-test. To asses normality, the Shapiro-Wilk test was used alongside graphical representations, including the Normal Q-Q plot, Detrended Normal Q-Q plot, and Box Plot. All the statistical analysis was performed using the Statistical Package for the Social Sciences (SPSS for Windows, version 18.0, Chicago III). The significance level was set at 5%. Beyond significance testing (*p*-value), effect size was also considered to evaluate the magnitude of the effect.

### Power analysis

Power analysis was performed using the open source software G*Power (3.1.9.6) and was used to calculated the minimum number of participants required to achieve reasonable power (>80%). A Post-hoc analysis revealed that in one of the main parameters related to the aims of the study (Aquaticity Score) we had enough power to detect statistical significant differences between the pre and post intervention comparisons [within groups aquaticity score: Effect size dz = 1.7312841, total sample size = 9, t = 2.2621572, Power (1-β err prob) = 0.9979393].

## Results

Participants’ basic characteristics are presented in Table 5. Briefly, participants were matched for gender, age and aquaticity levels before the randomization process. Even though height and weight differ statistically, BMI did not show any statistical differences between the two groups.

**Table 5:** Basic characteristics of the participants divided in two groups.

Parameters	Group A (swimming)	Group B (Aquaticity)	*P* value
*N*	10	10	–
Gender (M/F)	4/6	4/6	–
Age (yrs)	16.3 ± 0.8	16.8 ± 0.5	0.105
Height (cm)	167 ± 0.1	174 ± 0.1	**0** **.** **030** *
Weight (kg)	60 ± 6.9	70 ± 11.9	**0**.033*
BMI (kg/m^2^)	21.4 ± 1.1	22.9 ± 2.2	0.072
Aquaticity (AU)	29.1 ± 3.3	27.1 ± 3.8	0.239

BMI, body mass index.

* Differences between the two groups. Data are Mean ± SD.

Bold indicates statistical significant values.

Changes in Aquaticity score before and after the 2 months intervention program are presented in Table 6. Aquaticity score improved by 13.2% in Swimming (Group A) and 26.6% in Aquaticity (Group B), (*p* < 0.01) and the Delta change differences, in pre—post score, was 2 folds increased in the Aquaticity group compared to Swimming one (*p* = 0.001) implying higher magnitude of improvement in the aquaticity group.

**Table 6:** Changes in aquaticity score before and after the 2 months intervention.

Intervention	Group A (swimming)	Group B (aquaticity)	*P* value* (effect size)
Pre Training	29.13 ± 3.27^a^	27.17 ± 3.89^a^	0.239
Post Training	32.8 ± 2.28	34.08 ± 2.6	0.257
Delta Change	−3.67 ± 1.33	−6.91 ± 2.14	**0.001** (−0.253)
(%)	(−13.23 ± 6.88%)	(−26.6 ± 10.40%)^a^	**0.004** (0.604)

^a^ Differences within groups (Pre vs. Post), p,0.01. Data are Mean ± SD.

* Differences between the two groups.

Bold indicates statistical significant values.

Individual task's in the aquaticity test, before and after the 2 months intervention, is presented in Table 7. In Group A (swimming), 7 out of 10 tasks were improved significantly compared the pre-values (*p* < 0.05) while in Group B (aquaticity) 10 out of 10 shown significant improvements compared to pre-training values. Interestingly, the magnitude of change (Delta change) between the two groups was statistically significant in 5 out of 10 tasks (tasks 2, 3, 7, 9, 10) implying again higher magnitude of improvements in the aquaticity intervention group compare to swimming group where, only task-5, has shown larger magnitude of change compare to aquaticity one (Table 5).

**Table 7:** Individual task's aquaticity score before and after the 2 months intervention.

Tasks	Group A pre	Group A post	*P* value* (effect size)	Group B pre	Group B post	*P* value* (effect size)
Task 1 Δ change (%)	3.05 ± 0.59	3.50 ± 0.52 16.78 ± 16.1	**.004** (−0.375)	3.45 ± 0.64	4.3 ± 0.25 27.61 ± 18.54	**.001** (−0.658)
Task 2 Δ change (%)	3.80 ± 0.42	4.10 ± 0.21 9.16 ± 13.72	**.050** (−0.411)	3.50 ± 0.66	4.3 ± 0.34 25.27 ± 15.28^a^	**.001** (−0.606)
Task 3 Δ change (%)	3.20 ± 0.25	4.00 ± 0.00 25.71 ± 9.83	**.001** (−0.914)	2.85 ± 0.66	4.15 ± 0.33 50.41 ± 24.91^a^	**.001** (−0.779)
Task 4 Δ change (%)	3.55 ± 0.43	4.20 ± 0.25 19.28 ± 9.72	**.001** (−0.678)	3.25 ± 0.42	3.65 ± 0.47 13.09 ± 15.27	**.022** (−0.409)
Task 5 Δ change (%)	3.50 ± 0.40	4.0 ± 0.40 14.46 ± 1.70^a^	**.001** (−0.529)	3.05 ± 0.49	3.25 ± 0.35 7.66 ± 9.94	**.037** (−0.228)
Task 6 Δ change (%)	3.20 ± 0.78	3.30 ± 0.82 3.33 ± 10.54	.343 (−0.062)	3.40 ± 0.51	3.80 ± 4.22 13.33 ± 17.21	**.037** (−0.06)
Task 7 Δ change (%)	3.2 ± 0.26	3.5 ± 0.00 8.33 ± 8.78	**.015** (−0.632)	2.6 ± 0.45	3.55 ± 0.15 39.76 ± 21.60^a^	**.001** (−0.816)
Task 8 Δ change (%)	2.95 ± 0.68	3.30 ± 0.48 15.33 ± 21.49	**.045** (−0.285)	2.85 ± 0.24	3.70 ± 0.48 30.66 ± 20.41	**.001** (−0.745)
Task 9 Δ change (%)	2.08 ± 0.30	2.2 ± 0.26 7.01 ± 14.77	.239 (−0.209)	1.62 ± 0.38	2.18 ± 0.25 38.92 ± 24.97^a^	**.001** (−0.656)
Task 10 Δ change (%)	0.55 ± 0.15	0.70 ± 0.25 30.00 ± 48.30	.081 (−0.341)	0.60 ± 0.21	1.20 ± 0.42 100.00 ± 0,00^a^	**.001** (−0.670)

Group A swimming, Group B aquaticity.

^a^ Differences between delta changes *p* ≤ 0.05.

* Differences within group (pre vs. post).

Bold indicates statistical significant values.

## Discussion

This study addresses the aspect of ‘developing human aquaticity’ and how it can be improved using a structured training program in confined waters. The current study has shown that aquaticity score can be improved in healthy adolescents using structured and specific to aquaticity training regimes, compared to classical swimming training. This is the first study to show that aquaticity level can also be improved in healthy adolescents by a classical swimming program (13%) but significantly less compared to specific aquaticity training (27%).

The aquaticity score was acquired using the Aquaticity Assessment Test (8). In Group A where classical swimming training was used as an intervention, 3 out of 10 tasks did not statistically change after the intervention program (tasks 6, 9, 10). More specific, task 6 is related to treading water—egg beater kick, which is mainly used in specific aquatic sports like water polo, synchronized swimming and in lifesaving activities for maintaining a hands-free support of the body, by holding a vertical and stable position (11, 14, 15). This type of exercise is not commonly trained in classical swimming programs since it is not part of the technique of the competitive styles, that's why the egg- beater technique did not improve. Though trending water is mentioned as one of the stationary surface competencies, which is essential in a drowning scenario (14). Our findings are in agreement with Brenner et al. (16) who recommended that “swimming ability is a necessary component of water competence, but with the understanding that swimming ability alone is (often) not sufficient to prevent drowning”. In contrast task-6 was significantly improved in Group B as a result of the specific type of training. Similarly, task 9 which is related to breath hold underwater swimming- did not change, in Swimming Group A. Breath holding and underwater swimming is a common type of training in freediving, synchronized swimming, lifesaving sports and not in classical swimming. In contrast, the Aquaticity Group B, showed almost 40% improvement after the aquaticity program and statistically significant differences in the magnitude of change between the two groups. Since aquaticity is related to underwater performance it is expected that breath hold activities will be incorporated in the aquaticity training and therefore to be improved after the intervention training period. Task-10, which is considered to be one of the most challenging tasks of the Aquaticity test since it's required a voluntary exhalation and unsupported immersion in deep waters towards the bottom, control of underwater buoyancy and orientation, and a “goal achieving task in the maximum depth”, then regain the surface and approach the wall of the pool. This task is a simulation of a distressed situation that an individual could face in the water. One of the many examples of such incidents, is the immersion after involuntary entry in deep water, where according to Stallman et al. (17) this is a life-threatening incident. The performance in this task, did not change significantly after the conventional swimming training in the Swimming group, while improved 100% in the Aquaticity group. These types of exercises are part of free diving training as they promote the effective interaction of multiple skills and competences, self-control during breath hold, and also mental clarity and clear judgment during a worst-case scenario. In conclusion, task-10 is a “key challenge” that shows a person's behavioral response to a distressed situation in the water by having an anxiety attack and quitting the task or by showing willingness to face the task. Thus, aquaticity training program agrees with the recommendations of Stallman et al. (14) that “realistic emergency situations should be taught and combined in creative ways, challenging the learners “capability to cope with”.

**Figure 1:**
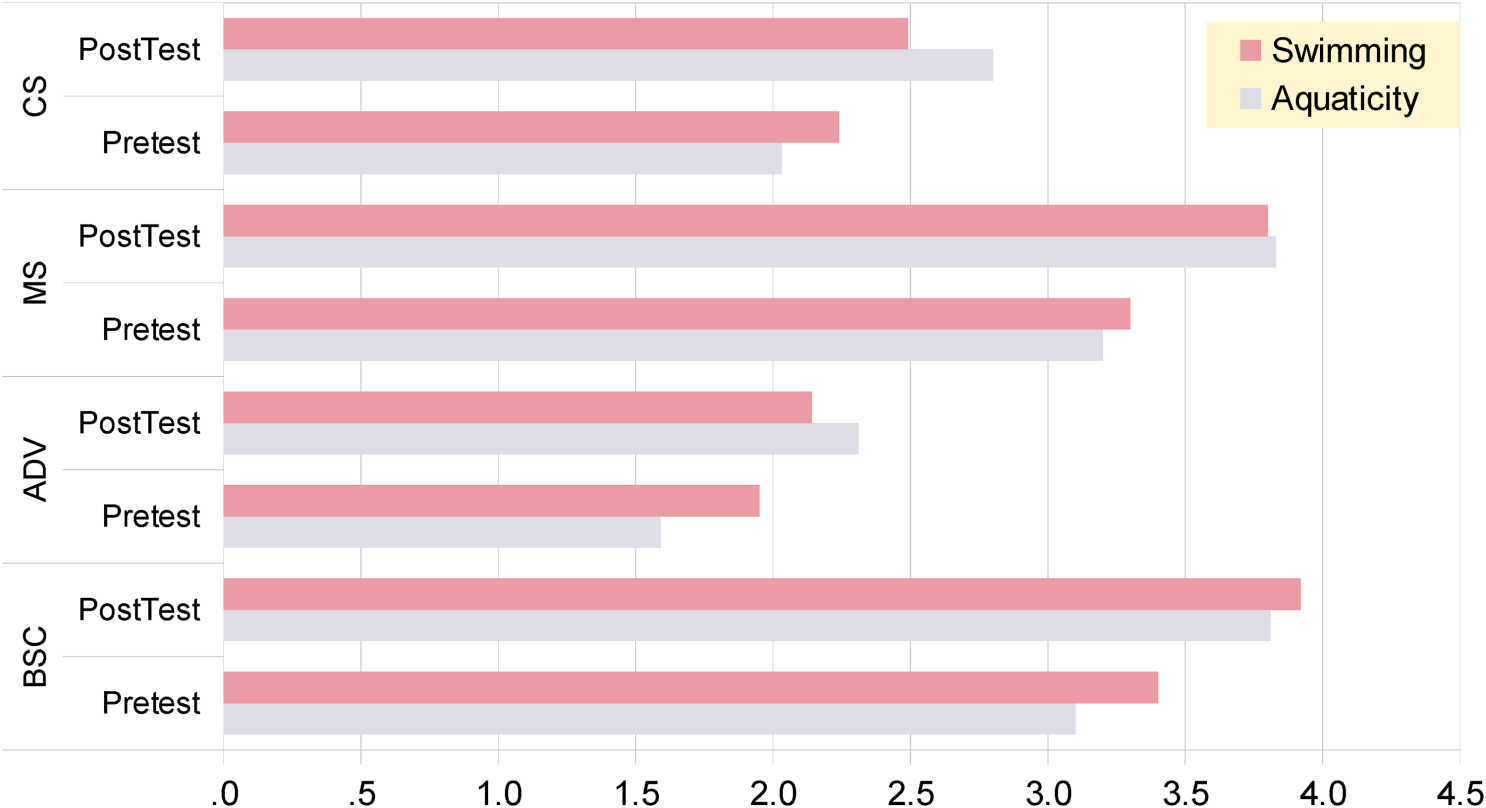
Means on improvement per group in pretest and posttest phase: breathing control (BSC, basic; ADV, advanced), and on cognitive-perceptual (CS) or motor (MS) skills, swimming vs. aquaticity group.

According to the categorization of tasks, we can see that task, in which Advance Breathing Control and Cognitive- Perceptual Skills interacted (tasks 7,9,10), showed a higher magnitude of improvements in the aquaticity program compared to the classical swimming program. The scores of the participants in the Advance Breathing Control, between Pre and Post measurements was almost double improved in favor of the Aquaticity group while the so-called Cognitive—Perceptual skills had also double improvement in Aquaticity vs. Swimming group (Figure 1). In addition, the score in the so-called Motor Skills, were found to be statistically significantly improved, after the classical swimming training by 26.2% compared to the Aquaticity group. Especially, task-5, has shown a larger magnitude of change in Swimming group compared to the Aquaticity group. This is expected since classical swimming training is based on improving swimming long distances and task-5 is related to physical adequacy and included a 5 min “any style surface swimming” to reach maximum distance possible. On the other hand, task-7, which was categorized in the Cognitive—Perceptual skills, is related to underwater vision and combines senses adaptability, together with advance breathing control and problem solving was improved in aquaticity group more than in the classical swimming. This ability can be trained and improved by frequent practice in the water without goggles or mask (18). Due to the fact that classical swimming uses goggles for training eliminates any training effect in this capability compared to the Aquaticity group where part of the training was taking place without any facial equipment. More specifically, in aquaticity program, students trained in visual scanning, finding and lifting submerged objects and also other creative underwater exercises without the use of goggles.

In the current study some potential strengths and weaknesses have been recognized that need to be acknowledged. The main strengths of the current study are the insights it offers into how a short-duration training program can significantly improve factors related to physical activity and well-being in healthy adolescents. Additionally, this study provided valuable insights into how humans can easily adapt underwater, as the aquaticity development program incorporated underwater tasks which showed significant improvement in post-intervention measurements. Another strength of this research is that it clearly showed that aquatic competencies can be significantly developed with a non-competitive specialized aquatic program.

Regarding the pitfalls that took place in the current study, firstly, the number of participants per group was limited to 10. A larger number of participants could have shed more light on the explanation of these findings. In addition, the study had only two groups for comparison. Additional groups from other aquatic sports such as synchronized swimming or freediving could have helped us explain our findings better.

Future research will show how aquaticity development programs could become an integrated aquatic method to improve specific characteristics of performance in competitive sports and also develop specific skills and cognitive-perceptual function during “a worst-case scenario” in a stressed swimmer or in military and rescuers training.

## Conclusion

Aquaticity is another parameter of human performance that can be trained and improved not only by classical swimming training but significantly more by specific aquaticity training. The Aquaticity development program improves aquatic efficiency not only on the surface but also underwater. The aquaticity program effectively developed aquatic motor and cognitive-perceptual skills, breath holding and senses adaptability, which all are essential for an individual when has to consider multiple variables simultaneously during a distressed situation in the water. Several aspects of behavior in the aquatic environment during a challenging task, including accuracy of performance, emotional stability, concentrated thinking, clear judgement, willingness to try and problem solving underwater, received the most benefit from the aquaticity program. Aquaticity training could be an alternative form of exercise for people who are not interested in competitive or ‘boring and repetitive training sets’ in swimming sport, as well as for those who need to reach a specific level of aquatic performance to participate in certain types of aquatic activities. Aquaticity training is effective as an educational intervention in school PE programs, promoting water competence in teenagers and encouraging physical activity without the stress of competitive swimming. Aquaticity programs could also be applied in Drowning Prevention, in aquaphobia therapy, in rehabilitation and also in hospitality, summer camps and in water sports clubs to improve performance and enjoyment in aquatic activities.

## Data Availability

The raw data supporting the conclusions of this article will be made available by the authors, without undue reservation.
